# A review of finite element modeling and surgical simulation of meniscal tear in knee joint: progress and challenges

**DOI:** 10.3389/fmed.2025.1661943

**Published:** 2025-09-12

**Authors:** Chengyue Yu, Xiaoyuan Duan, Yu Gou, Kexin Liu, Wenjun Zhao, Xiaokang Gao, Lupeng Wang, Jinwei Liu, Jiahe Xu, Jiashi Zeng, Desheng Chen, Guosheng Xing, Weiguo Xu

**Affiliations:** ^1^Tianjin Hospital, Tianjin University, Tianjin, China; ^2^Weifang People's Hospital, Weifang, China; ^3^Department of Orthopedics, North China Medical Health Group Fengfeng General Hospital, Heibei, China; ^4^Clinical School/ College of Orthopedics, Tianjin Medical University, Tianjin, China

**Keywords:** knee joint modeling, finite element analysis, meniscal tears, meniscectomy and repair, clinical analysis

## Abstract

As one of the essential tissues of the knee joint, the meniscus plays a crucial role in load transmission, shock absorption and joint stability. Meniscal tears caused by degenerative diseases and traumatic injuries are prevalent. Meniscal repair or meniscectomy is considered the first choice for treatment. Because the knee joint cannot be conducted *in vivo*, and the reproducibility of *in vitro* experiments is poor, finite element analysis has become an important tool for evaluating clinical surgical techniques. This review summarizes the latest research progress on meniscal tears and corresponding surgical techniques from the perspective of numerical calculation and clinical analysis for the first time. The study found that establishing an accurate finite element model requires consideration of multiple factors and rigorous clinical validation. The purpose of this review is to provide researchers with more reasonable finite element models, evaluate the biomechanical characteristics of meniscal tears and related surgical techniques, and provide more systematic research for clinical practice to improve surgical techniques further. This presents new research opportunities for the precise diagnosis and treatment of knee joint diseases.

## 1 Introduction

The knee joint is one of the most complex joints in the human body and plays an important role in normal human activities (1, 2). Because of long load-bearing times and a large amount of exercise, the knee joint is prone to osteoarthritis (OA) (3). There are many causes of OA, among which meniscus injury is one of the important factors. The meniscus is a semicircular structure composed of fibrocartilage that acts as a load sharer and shock absorber in the knee joint (2, 4, 5). Degenerative changes and traumatic injuries can induce meniscal damage, leading to disruption of collagen fiber networks and subsequent deterioration of biomechanical properties, which predispose to pathological meniscal extrusion (6). Meniscal extrusion (ME) has been strongly associated with cartilage wear and osteoarthritis (OA) progression (7–9). Furthermore, meniscal injuries often compromise joint stability through secondary structural damage—for instance, posterior root tears frequently coincide with ligamentous laxity or concomitant ligament injuries. Once a meniscal tear occurs, it is difficult to heal on its own unless it happens in the red zone rich in capillaries (10). The main methods for treating meniscal tears currently used are meniscectomy (partial, subtotal or total) and meniscal suturing (11, 12). This surgery relieves the patient’s pain and prevents joint inflammation by removing or suturing the tearing meniscus. Based on the patient’s condition and the experience of surgeons, meniscus repair has become the first choice for treatment.

To better understand the structure and function of the meniscus, researchers have employed finite element analysis to investigate the mechanical response of intra-meniscal tissue. This approach provides mechanistic explanations for clinically observed phenomena. Finite element analysis greatly reduces experimental costs and improves experimental efficiency (1, 13, 14). It also enables more comprehensive data analysis. At present, significant progress has been made in the numerical analysis of different types of menisci tears and corresponding surgical techniques, which has attracted widespread attention from researchers. This review elaborates on the “Introduction,” “Finite element modeling of meniscal tear of knee joint,” “Finite element analysis of different types of tears and surgery of the meniscus,” “Discussion and future perspectives” and “Conclusion.” This study focuses on the establishment of finite element models of knee meniscal tears, providing researchers with more reasonable finite element models. Furthermore, this study aims to evaluate the biomechanical characteristics of meniscal tears and related surgical techniques, offering systematic insights for clinical applications and ultimately improving surgical techniques.

## 2 Methods

**Figure 1:**
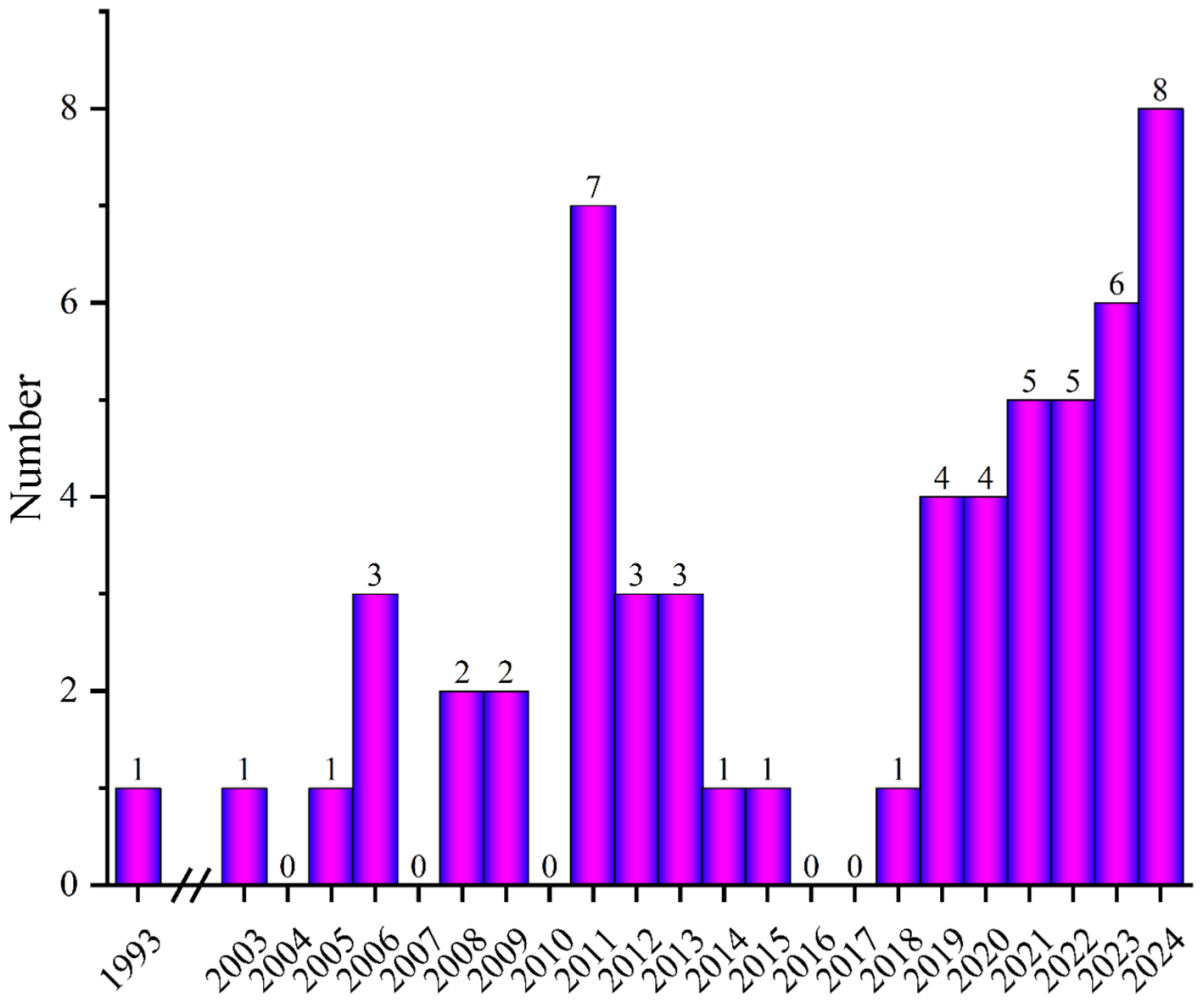
Number of publications related to “finite element modeling of meniscal tear” per year.

A literature search was conducted using search engines such as Google Scholar, PubMed, and WOS, focusing on publications related to finite element modeling and simulation of knee meniscus. Figure 1 shows the number of publications related to “Finite element modeling of meniscal tears” per year. Based on the abstracts and content of the papers, papers that met the following three criteria were selected: (1) three-dimensional finite element model of the knee joint; (2) simulation of meniscal tears and surgical techniques; and (3) material analysis and wear research of knee meniscus. Exclusion criteria: (1) non-English articles; (2) titles and abstracts that do not match; and (3) low-quality articles (Whether to validation of model validity).

Given the narrative design of this review, the study also did not follow a pre-defined registered protocol or systematic review standards such as those outlined in the PRISMA guidelines.

## 3 Finite element modeling of meniscal tear of knee joint

**Figure 2:**
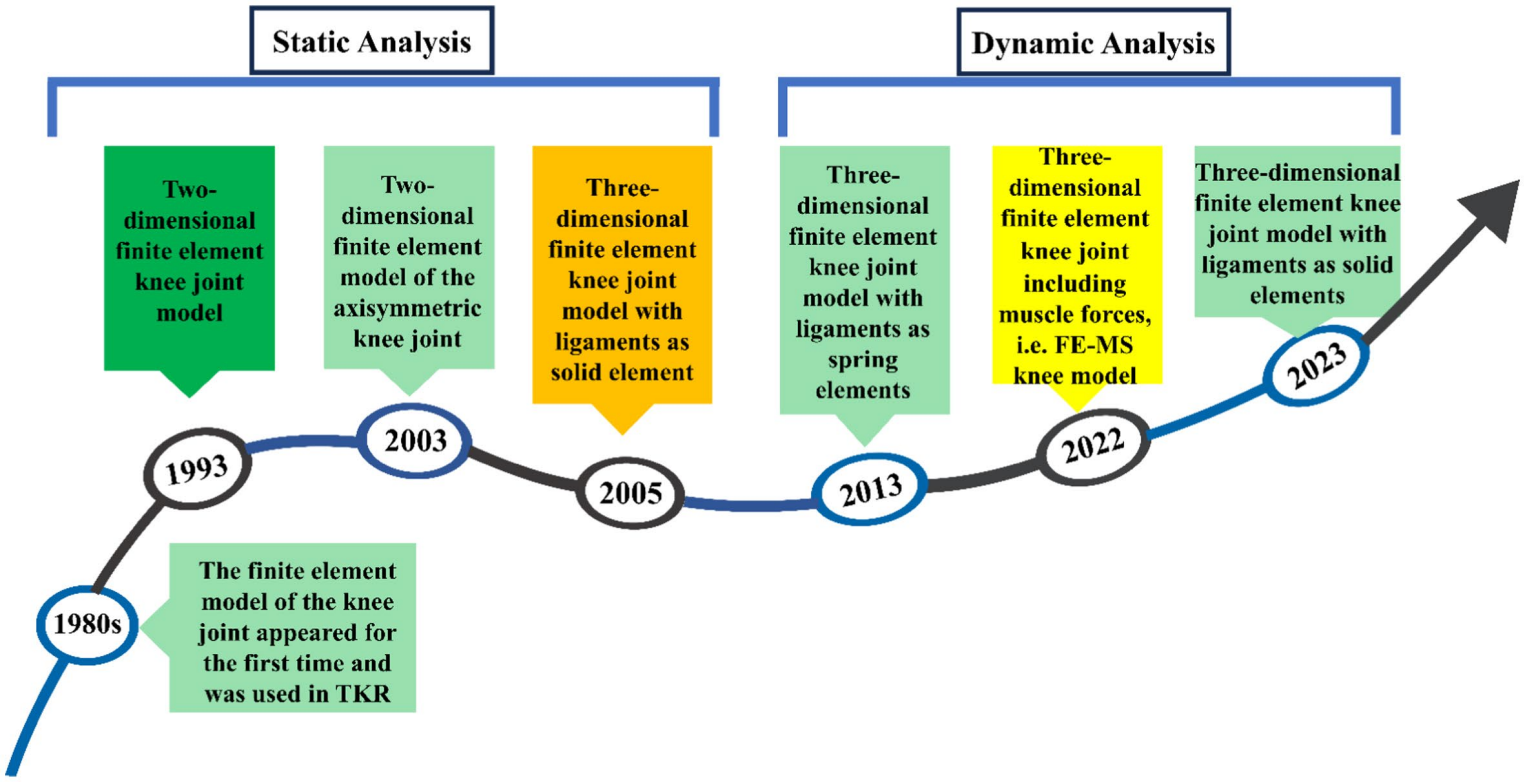
Development of the finite element model of knee meniscal tears.

Figure 2 shows the development process of finite element modeling of the meniscal tear knee joint, mainly involving several key time nodes and the evolution of the model. Finite element analysis (FEA) was first introduced to orthopaedic biomechanics in the 1980s, with early applications primarily focused on the optimization of prosthetic design in total knee arthroplasty (TKA) (15). In 1993, the first two-dimensional (2D) finite element model of the knee joint was developed to investigate the biomechanical effects of meniscectomy (16). With advances in computational power, three-dimensional (3D) static knee models emerged in the early 2000s (17), evolving into dynamic gait cycle analyses post-2010 (18). By 2022, the field had progressed to integrated finite element-musculoskeletal (FE-MS) modeling, enabling simulation of joint responses under physiological loading conditions (12).

### 3.1 Knee joint modeling and analysis process

**Figure 3:**
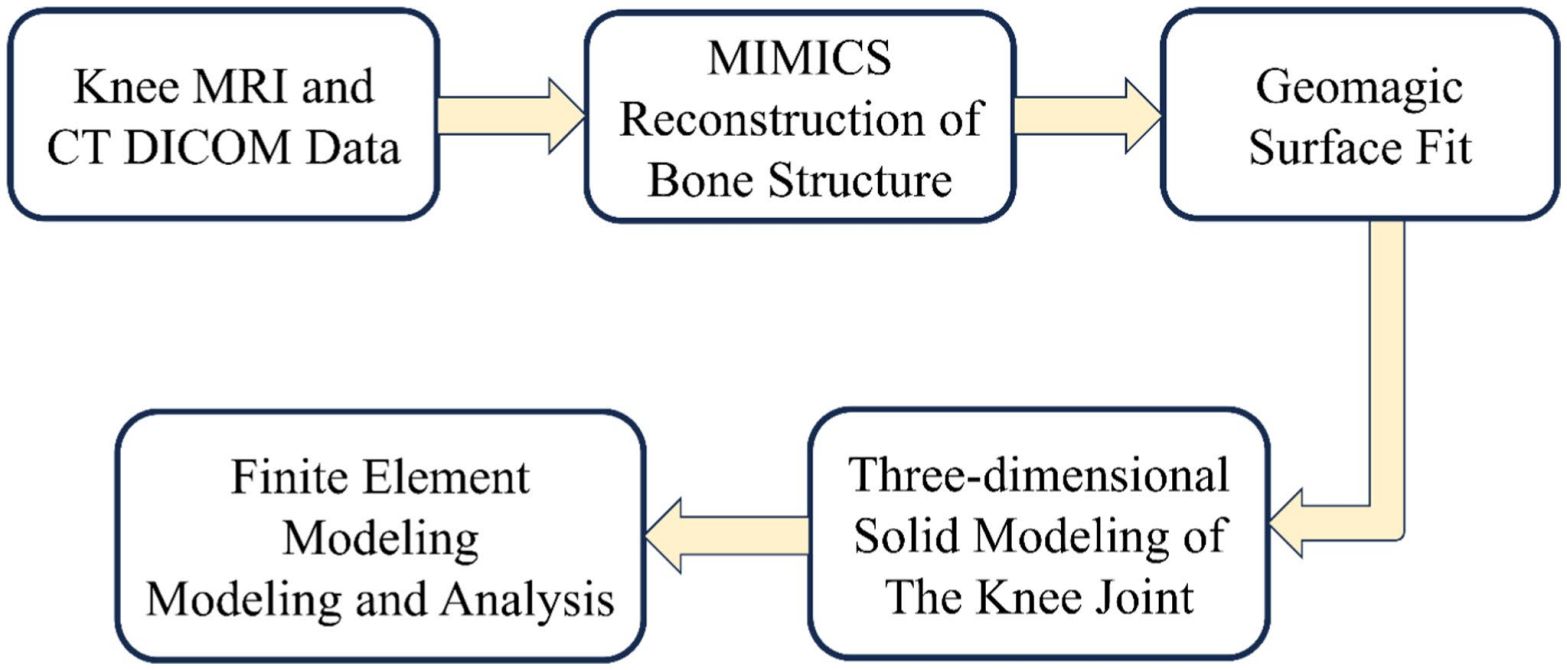
Flow of knee joint modeling and analysis.

The development of knee joint models involves two critical phases: geometric modeling and finite element modeling. The establishment of geometric modeling begins with the acquisition of medical images [magnetic resonance imaging (MRI) and computed tomography (CT)]. These medical images undergo high-resolution scanning to enhance knee joint geometric accuracy. Establishing the finite element model includes meshing, material property assignment, contact setting, and boundary condition setting. Mesh refinement is critical to improve analysis accuracy, though it requires balancing computational cost. The process is shown in Figure 3:

Finite element analysis of the knee joint remains both a research priority and a technical challenge in this field. It mainly focuses on meshing and boundary condition settings. For 3D finite element models, hexahedral or tetrahedral elements are typically employed for mesh generation. Hexahedral meshes have higher accuracy. However, due to the existence of the knee joint surface, hexahedral meshes cannot be automatically generated, and the difficulty of meshing increases. Benos et al. (1) demonstrate that the automatic generation of hexahedral meshes is still a challenging research point. On the contrary, the advantages of tetrahedral meshes are demonstrated here. It can handle the meshing of complex surfaces and has high analysis accuracy. It has become the preferred mesh for researchers. Tetrahedral element types commonly used in finite element analysis include linear tetrahedra (C3D4), quadratic tetrahedra (C3D10), and modified quadratic tetrahedra (C3D10M). Among these, C3D4 elements exhibit the lowest numerical accuracy, whereas both C3D10 and C3D10M elements provide superior accuracy. The C3D10M element is particularly widely employed in finite element analyses of the knee joint due to its enhanced performance in modeling nonlinear material behavior and large deformations, thereby effectively mitigating potential volumetric locking issues. But tetrahedral meshes are only suitable for static analysis. High-precision hexahedral meshes are often the first choice for dynamic analysis. Current computational studies face significant challenges in performing dynamic analyses of intact knee joints, primarily due to the large deformation characteristics of ligaments that complicate accurate biomechanical simulations. The contact settings in the boundary conditions usually affect the convergence of the analysis. The author will describe it in detail in the “Simulation loads and boundary conditions” section.

Nonlinear problems have always existed in finite element analysis, affecting the convergence of the results. They mainly include material nonlinearity, geometric nonlinearity, and boundary nonlinearity. Researchers now solve the nonlinear problem of knee joint analysis by dividing high-quality meshes and setting reasonable contact conditions. Generally speaking, the inherent anatomical variability of knee joint structures among individuals results in non-generalizable computational models, necessitating rigorous validation of finite element analyses. The author will describe it in detail in the “Validation of model validity” section.

### 3.2 Simulation loads and boundary conditions

In the finite element analysis of the knee joint, the contact is usually defined according to the actual situation. Researchers have different views on the contact properties of these structures. Xu et al. (11) demonstrated that synovial fluid reduces cartilage friction. However, it has never achieved absolute smoothness. The friction coefficient between soft tissues is 0.002, representing a hard contact behavior. Other researchers (19) believe that the contact between soft tissues allows tangential sliding with a friction coefficient of 0.02. However, some researchers, such as Bae (20) and Pena (21), believe that the contact surfaces are all frictionless nonlinear contacts. Despite these variations, Rooks et al. (22) established through parameter sensitivity analysis that the penalty function exhibits negligible influence on outcome validity. Thus, while contact definitions differ, their impact on conclusions remains marginal. In the finite element modeling of knee joint contact, it is essential to properly define contact attributes for both the initial configuration and potential contact interactions during loading phases to prevent mesh penetration and ensure computational convergence.

**Figure 4:**
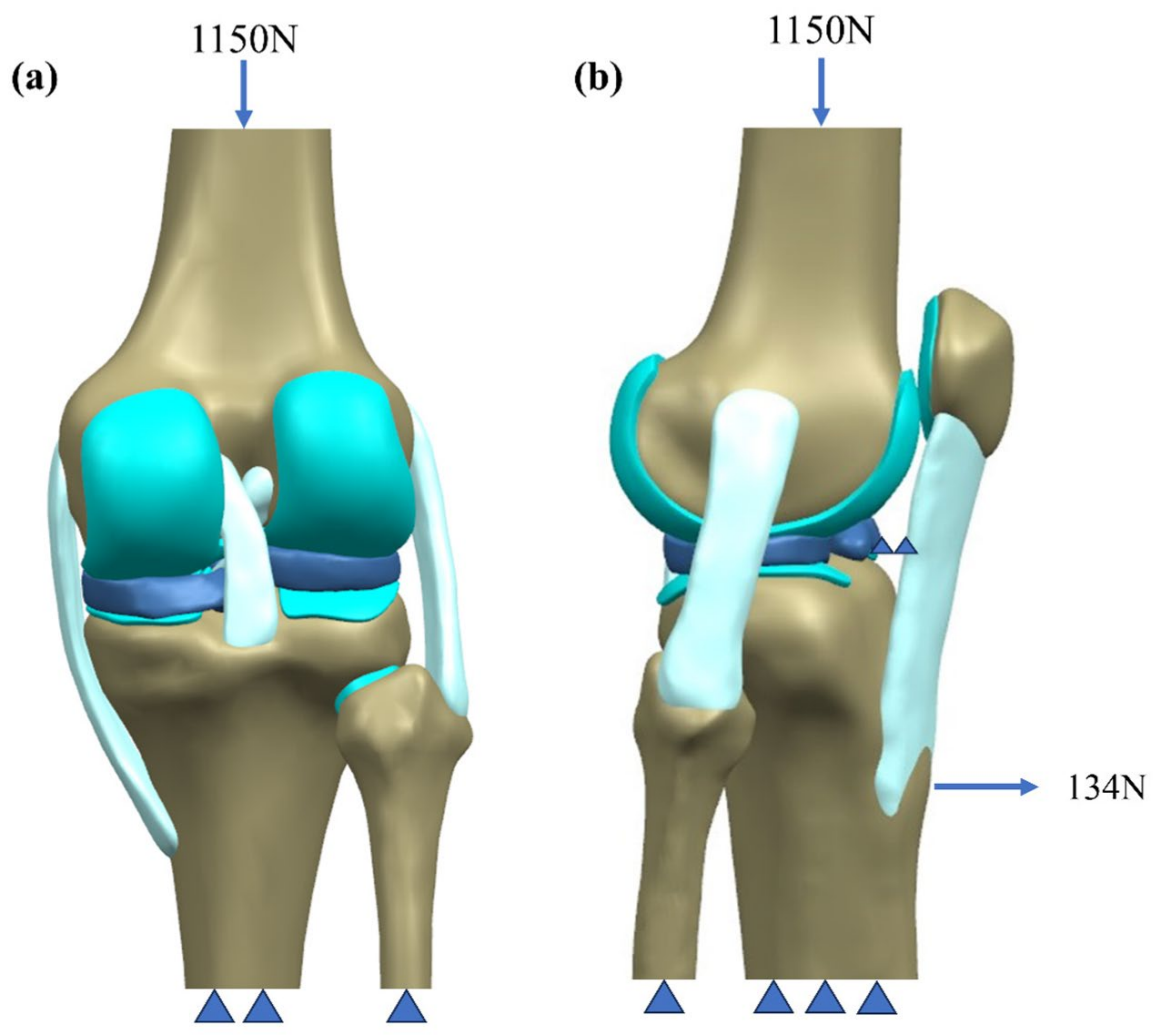
Schematic of boundary conditions. **(a)** Static attitude simulation. **(b)** Slight buckling simulation.

**Figure 5:**
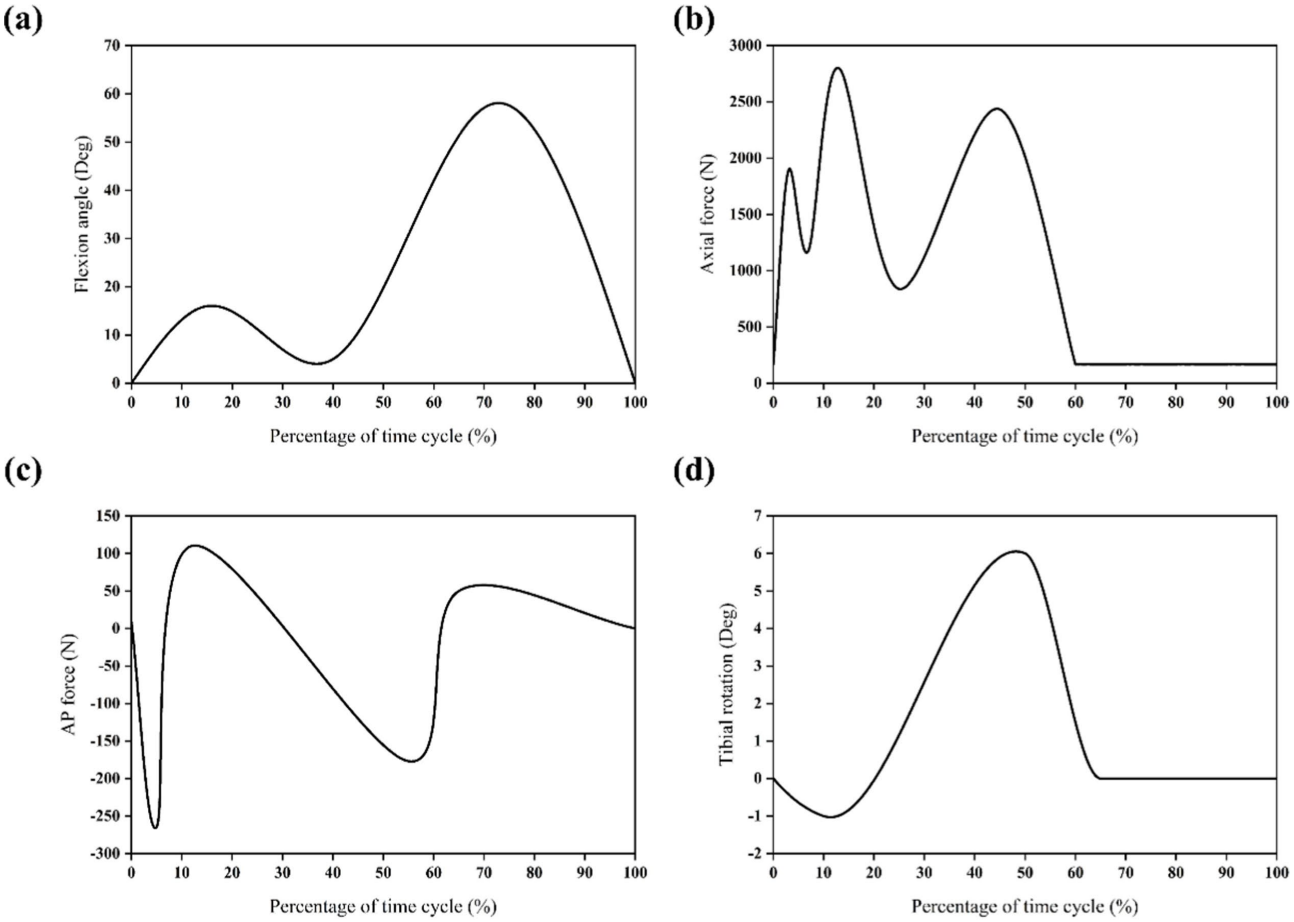
Input function of finite element model based on ISO 14243-1 gait cycle. **(a)** Flextion angle. **(b)** Axial force. **(c)** AP force. **(d)** Tibial rotation.

**Figure 6:**
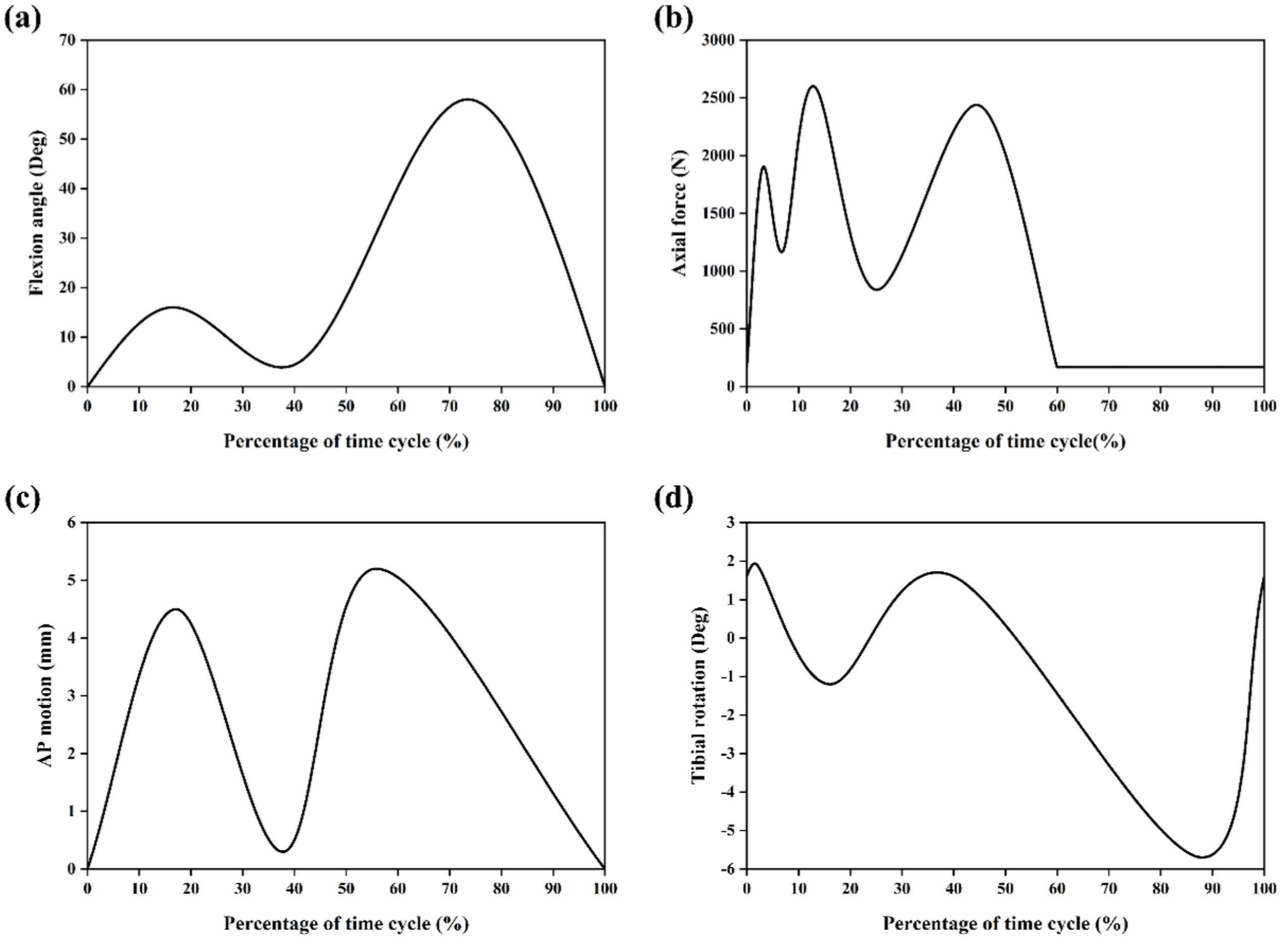
Input function of finite element model based on ISO 14243-3 gait cycle (11). **(a)** Flextion angle. **(b)** Axial force. **(c)** AP motion. **(d)** Tibial rotation.

In simulations of meniscal tears and meniscectomy, the applied loading and boundary conditions vary depending on research objectives, primarily categorized into two types. The first type is static analysis, which is the most widely used. All translations and rotations of the tibia and fibula are fixed. A vertical pressure of 1,150 N (twice the body weight) is applied to the upper surface of the femur (static posture simulation) to simulate the force of the gait cycle in the extended position (21, 23–28) as shown in Figure 4. The second type is dynamic analysis. Dynamic analysis can better simulate the cyclical motion of the knee joint in real life. Based on the guidelines of the International Organization for Standardization (ISO 14243-1 and ISO 14243-3) as the input of dynamic simulation, to simulate the entire gait cycle of knee motion, as shown in Figure 5 and Figure 6. Yang et al. (27) demonstrate the dynamic simulation after the posterior root tear and posterior horn resection of the medial meniscus of the knee joint—the only published dynamic FEA of intact knee joints (including ligaments). This is the only literature that the author has consulted that uses a complete knee joint (including ligaments) for dynamic analysis. Dynamic analysis is often challenging because of the complex model calculation.

### 3.3 FE-MS model of knee joint

**Figure 7:**
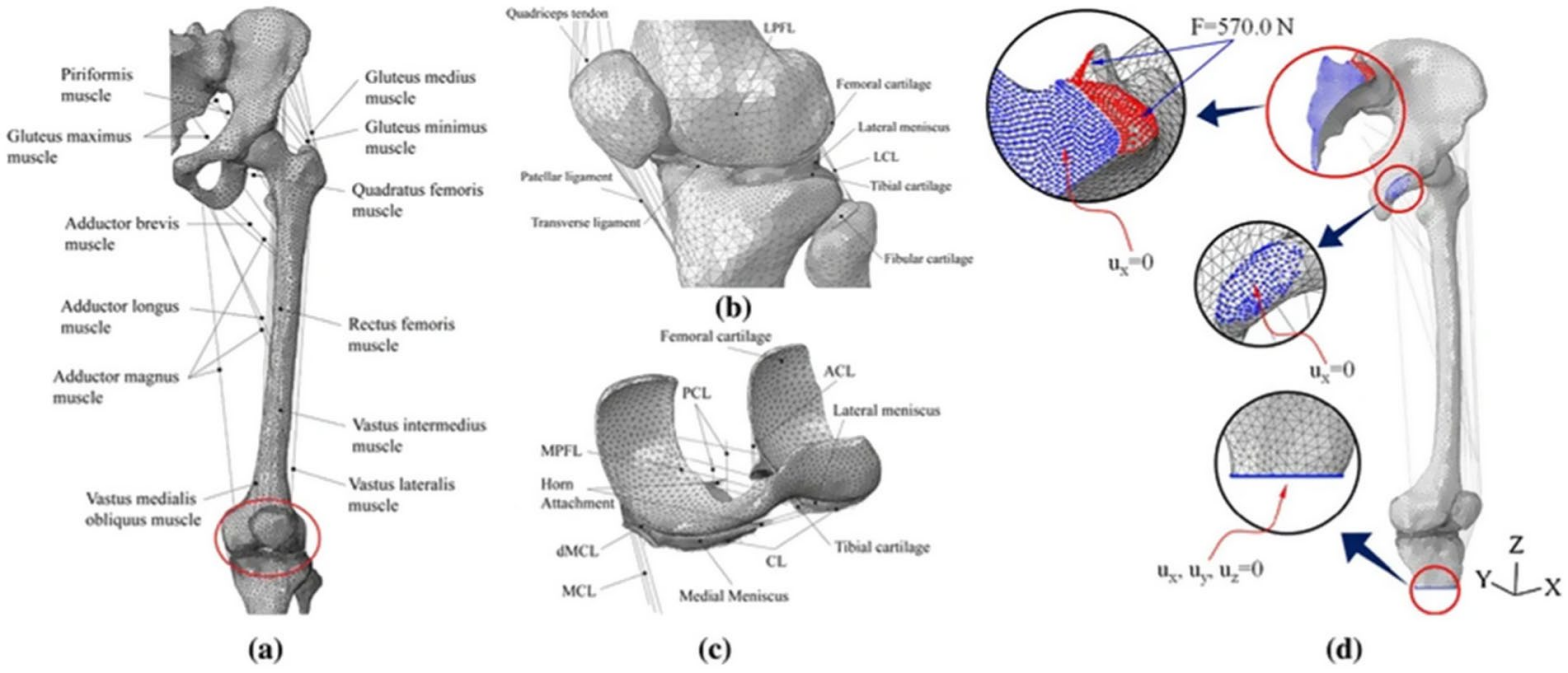
The finite element model of the lower limb was developed by Bae et al. (19). **(a)** Frontal view. **(b)** Enlarged view of tibiofemoral joint part. **(c)** Contact structure between cartilage and meniscus. **(d)** Boundary conditions for the computation.

**Figure 8:**
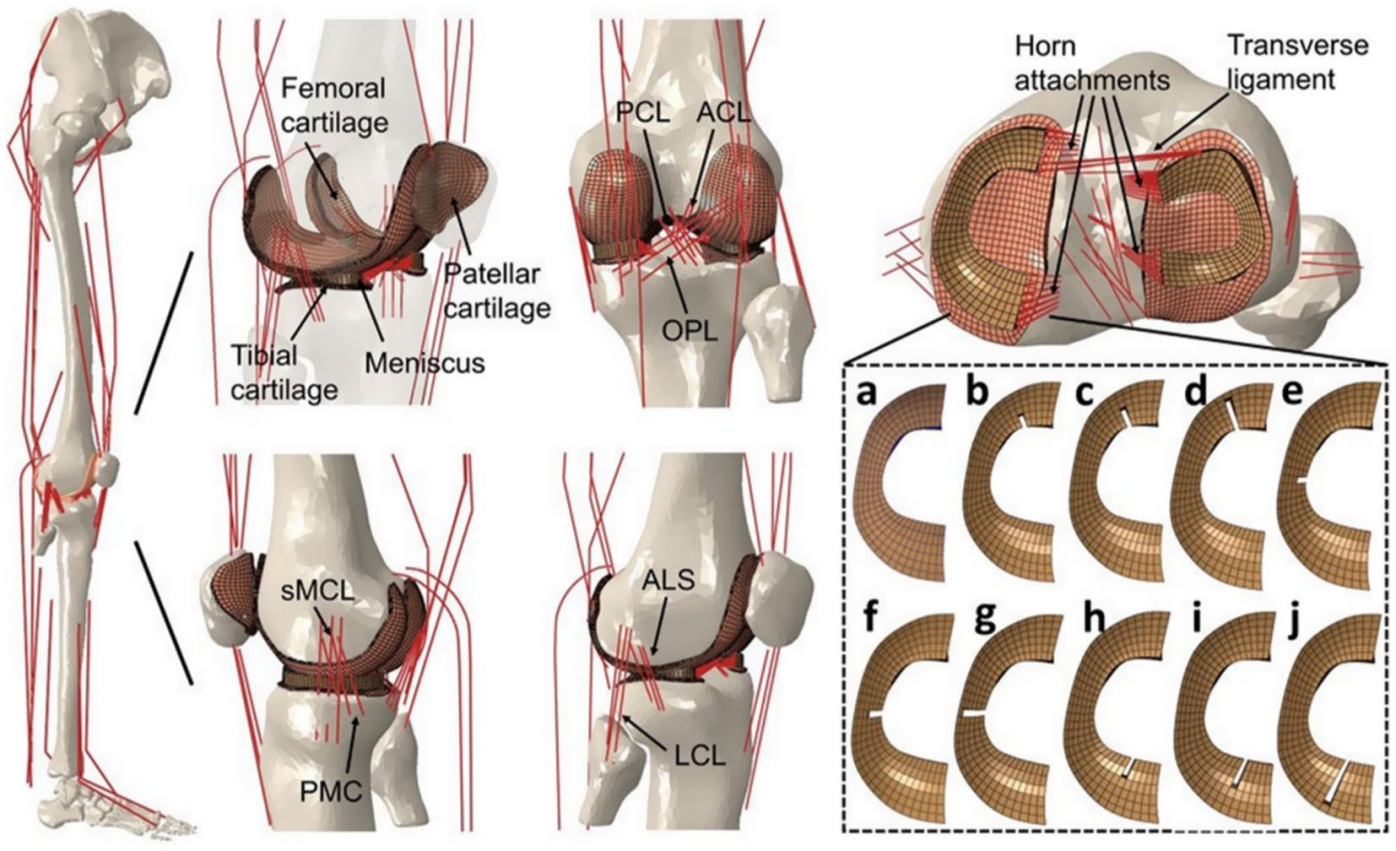
FE-MS model of the right lower extremity and a radial tear of the meniscus developed by Wang et al. (11).

The above biomechanical analysis of the knee joint was based on simplified load and boundary conditions (such as fixed compression load and flexion angle). These analyses fail to account for muscle-driven joint motion, prompting recent development of FE-MS models (29–33). The FE-MS model was first proposed and applied to the finite element analyses of total knee replacement (TKR) to improve its performance (33). Among the selected literature, Bae et al. (20) and Wang et al. (12) used the FE-MS model to analyze meniscal tear resection. Bae et al. (20) believed that partial meniscectomy could be considered a better treatment method than subtotal/total meniscectomy. Wang et al. (12) constructed an FE-MS lower limb model to study the biomechanical changes of radial tears of the medial meniscus caused by knee osteoarthritis (OA) during walking. The FE-MS model is shown in Figure 7 and Figure 8.

### 3.4 Material properties of knee meniscus

In addition to establishing accurate knee joint geometry and precisely characterizing the mechanical behavior of each tissue, appropriate material property assignment is critical for obtaining reliable analysis results. Material selection primarily considers the authentic mechanical properties of each tissue, with particular emphasis on meniscal material parameters in this review. Currently, there are five main meniscus material properties, which are isotropic elastic materials, transversely isotropic elastic materials, transversely isotropic hyperelastic meniscus material, fiber-reinforced poroelastic materials (FRPE), and biphasic materials.

Given the complexity of finite element analysis of the knee joint, researchers usually simplify the meniscus material into an isotropic linear elastic material (3, 17, 20, 24–28, 34–39). The elastic modulus E is 140 MPa, and the Poisson’s ratio ν is 0.45. However, linear elastic materials cannot represent the microstructure of the meniscus. Studies demonstrate that the meniscus is composed of water, collagen fibers, and matrix. Collagen fibers are distributed in a circumferential direction, and the meniscus has high stiffness in the fiber direction. Transversely isotropic materials are a better choice (2, 12, 19, 23, 40–48). The material parameters are shown in Table 1. Haut Donahue et al. (49) first used finite element analysis combined with contact pressure from knee joint experiments to conclude that meniscus material is a transversely isotropic linear elastic material, laying the foundation for the subsequent design of knee meniscus materials. Nevertheless, the transversely isotropic elastic (TIE) material model presents some limitations: (i) the intrinsic orthotropic structure of the tissue is simplified and (ii) the compression tension nonlinearity along the fiber direction and poroelastic properties are not considered (50).

**Table 1:** Meniscus material parameters (1).

Component	$E_{\theta }(\mathrm{MPa})$	$E_{r},E_{z}(\mathrm{MPa})$	$\nu_{\mathrm{r\theta}},\nu_{\mathrm{z\theta}}$	$\nu_{\mathrm{rz}}$	$G_{\mathrm{r\theta}},G_{\mathrm{z\theta}}(\mathrm{MPa})$	$G_{\mathrm{rz}}(\mathrm{MPa})$
Lateral meniscal	120	20	0.3	0.2	57.7	8.33
Medial meniscal	120	20	0.3	0.2	57.7	8.33

E, Young modulus; v, Poisson ratio; G, shear modulus. The subscripts θ , r , and z are circumferential, radial, and axial constants directions, respectively.

Transversely isotropic hyperelastic meniscus materials are currently rarely used (51). By combining the strain energy density function with the Holzapfel–Gasser–Ogden (HGO) material model, the function is constructed in the form of (11):


(1) $$U=C_{10}({\bar{I}}_{1}-3)+\frac{1}{D_{1}}(\frac{{\left(J_{\mathrm{el}}\right)}^{2}-1}{2}-\text{In}J_{\mathrm{el}})+\frac{k_{1}}{2k_{2}}(\exp[k_{2}{{\bar{E}}_{a}}^{2}]-1)$$

With


(2) $${\bar{E}}_{\alpha }=\kappa ({\bar{I}}_{1}-3)+(1-3\kappa )({\bar{I}}_{4(\alpha \alpha )}-1)$$

where ${\bar{I}}_{4(\alpha \alpha )}$ a pseudoinvariant of the symmetrically modified Cauchy-Green strain tensor, which simulates hard elastic collagen fibers. Among the parameters, $C_{10}$ , $D_{1}$ , $k_{1}$ , $k_{2}$ , and κ (Table 2) are used by Abaqus software to simulate real hyperelastic material properties in the calculation.

**Table 2:** Material parameters used for modeling the medial and lateral meniscus (10).

Component	$C_{10}(\mathrm{MPa})$	$D_{1}(\mathrm{MP}a^{-1})$	$k_{1}$	$k_{2}$	κ
Medial meniscus	1	5e^−3^	5.0	0.9	0
Lateral meniscus	1	5e^−3^	8.5	1.6	0

$C_{10}$ and $D_{1},$ neo-Hookean constants; $k_{1}$ and $k_{2}$ , HGO coefficients; κ , fiber dispersion and orientation level.

Due to its structural properties, the meniscus, like cartilage, can be treated as a FRPE material (52–55). A FRPE material considers a porous and hyperelastic media reinforced by collagen fibers, which can estimate the contribution of different constituents (collagen, proteoglycans, and fluid) on the mechanical response of the tissues. FRPE material can be applied to simulate transient and static loading conditions of the knee joint with the same material parameters (56–58), making the computational knee model of the meniscus more realistic. However, with the complex geometric model of the knee joint, implementing FRPE material is a difficult task.

The meniscus biphasic material model more comprehensively reflects the actual biomechanical behavior of the soft tissue of the knee joint (10, 55, 59, 60). The biphasic theory considers soft tissue to be a mixture of a porous permeable solid phase and an interstitial fluid phase (52). The theory shows that the fluid phase bears most of the load in a physiologically relevant short loading time (61, 62). The introduction of the biphasic theory solves the shortcoming that the soft tissue is simplified as an elastic material and only applies to transient responses. The biphasic model requires the compressive stiffness and Poisson’s ratio of the non-fibrillar matrix, tensile moduli of the collagen fibers and permeability to define the material properties (63). Berni et al. (55) first reported the regional and strain-related parameters of a fiber-reinforced biphasic model based on the characteristics of the human lateral meniscus.

In addition to the five material models described by the authors above, researchers have applied other meniscus material properties. Hauch et al. (64) obtained Young’s modulus of the meniscus attachment structure through tensile failure tests. The lateral anterior attachments were 161 MPa, the lateral posterior attachments were 96.3 MPa, the medial anterior attachments were 179 MPa, and the medial posterior attachments were 85.3 MPa. Therefore, Zielinska et al. (65) and Łuczkiewicz et al. (66) used linearly elastic springs to connect the nodes on the meniscus horns and the insertion points on the tibial surface to simulate the attachment. At each horn attachment, 10 linear springs with a stiffness of 200 N/mm were used to connect the tibial platform (67). Kedgley et al. (19) demonstrated that the insertion ligament at the attachment was modeled as a linear uncompressive spring. The tensile forces of the ligaments in the lateral anterior, lateral posterior, medial anterior, and medial posterior of the meniscus were 216, 130, 169, and 207 N/mm, respectively. Daszkiewicz et al. (43) also simulated the meniscal horn attachments using nonlinear spring elements that only bear tension. The stiffness of each meniscal horn attachment was assumed based on the experimental linear stiffness. Table 3 reports the meniscus horn attachment parameters. However, it has been reported in the literature that the use of spring elements significantly impacts the displacement compared to the 3D shape (68). De Rosa et al. (69) used a novel inverse finite element analysis method and showed that the average elastic modulus of collagen fibers was 287.5 ± 62.6 MPa. Tissakht et al. (70) conducted tensile tests on 31 human meniscus specimens and found that the elastic moduli of the anterior, middle, and posterior parts of the radial and circumferential specimens were 7.82, 11.49, 13.04 MPa and 99.75, 90.22, and 102.12 MPa, respectively.

**Table 3:** Parameters of meniscal horn attachments (47).

Meniscal horn	Lateral anterior	Lateral posterior	Medial anterior	Medial posterior
l(mm)	11.08	11.90	10. 78	6.79
$k_{h}(N/\mathrm{mm})$	253.50	106.6	218.0	218.7
N	55	85	67	64
$k_{s}(N/\mathrm{mm})$	4.609	1.254	3.254	3.417

$k_{h}$ , the total tensile stiffness; $k_{s}$ , the spring stiffness.

The meniscus has complex material properties (11, 40, 51, 64, 66, 67, 71), and its biomechanical parameters are difficult to define clearly. According to the review by Peters (72), the early data came from human samples and some researchers (73) also proposed based on the characteristics of canine menisci. Measuring the meniscus’s material properties may help identify degenerative human meniscus *in vivo* and be applied to other human soft tissues in the future (74).

### 3.5 Meniscus wear

Wear is considered to be the gradual removal of material from the working surface of an object due to the relative motion of the surfaces and is affected by the mechanical properties of the contacting materials, the working conditions, and the type of lubricant at the contact interface. The presence of lubricating fluid within the knee joint significantly reduces the wear rate of the meniscus under healthy conditions. In the current literature, numerous studies focus on cartilage wear and joint friction, while only a few investigate meniscal wear. A significant challenge in characterizing the wear behavior of the meniscus is the lack of established methods for measuring soft tissue wear parameters (75).

Meachim et al. (76) found through cadaveric studies that the fibers of the adult meniscus are susceptible to matrix wear, and mechanical factors play a significant role in the development of matrix wear. Moschella et al. (77) found that wear of the medial meniscus leads to accelerated cartilage wear. Severe joint deformity (a median 10° varus) has only 33.3% of intact meniscus, which indicates that meniscus wear is closely related to varus deformity. Bowland et al. (78) used optical measurement techniques to measure local volume loss in porcine menisci subjected to external joint loading. Benfield et al. (75) developed a 3D scanning method to quantify and visualize the wear behavior of the entire human meniscus tissue. The medial and lateral menisci lost approximately 60 and 55% of their volume, respectively, after 1 million load cycles. After 250,000 cycles, the volume wear rate of the medial meniscus leveled off at 0.72 cm^3^/Mc. Characterizing the wear behavior of the meniscus is crucial for understanding the pathological mechanisms of the disease and developing effective strategies to prevent, delay, and treat it. Cai et al. (79) showed that changes in the collagen microstructure when the meniscus was worn were the beginning of joint damage.

### 3.6 Validation of model validity

The accuracy of model establishment and result validation mainly depends on “geometric modeling,” “mesh-independent validation,” and “validity.” “Geometric modeling” is a necessary step in the finite element analysis of the knee joint. The high definition of CT and MRI images has ensured that the knee joint has a high-quality geometric model. “Mesh-independent verification” is an essential step in evaluating whether a model’s result is within the error range, which is the key to the self-correction of the model. Unfortunately, only a few researchers (20, 23, 43) have mentioned mesh-independent verification. Cooper et al. (13) mentioned different views on knee model validation, referring readers to their review. The gold standard for model validation is to test that model results continue to correspond well to the experimental data when testing independent samples. Knee validation has been a difficult problem for many researchers because of the inability to perform *in vivo* experiments. Currently, four validation approaches are widely adopted for knee joint finite element models: (1) Hertz contact theory analyses, (2) comparative experiments (2, 17, 21, 23, 25, 28, 40, 43, 66, 80–82), (3) cadaver experiments (43, 48, 83–95), and (4) gait experiments (96–101).

## 4 Finite element analysis of different types of tears and surgery of meniscus

### 4.1 Radial tear

#### 4.1.1 Radial tear of the body

Radial tears, extending perpendicularly from the meniscal edge toward the periphery, represent the most common clinical injury pattern associated with physical activity (102). Thus, investigating radial tears is critical for optimizing knee joint management. Clinical studies demonstrate that the medial meniscus sustains higher functional loads compared to the lateral meniscus, consequently exhibiting greater predisposition to injury. Dong et al. (23) analyzed radial tears in the medial meniscal body. They found that the peak compressive and shear stress of the femoral cartilage and tibial cartilage increased by about 20 and 70%, respectively, with medial meniscal stresses rising by approximately 50%. Meniscectomy shifted the peak pressure location, elevating medial meniscal compressive and shear stresses by 80 and 50%. However, studies have indicated that radial tears involving less than 50% of the meniscal width have no significant effect on tibial cartilage stress (103). Zhang et al. (25) further studied radial tears in the middle and posterior parts of the medial and lateral menisci and the corresponding meniscectomies. The study found that medial meniscal tears caused more significant stress than lateral ones, and meniscectomy markedly increased joint stress. These findings elucidated the mechanical mechanism of meniscectomy and are more likely to produce adverse effects.

**Figure 9:**
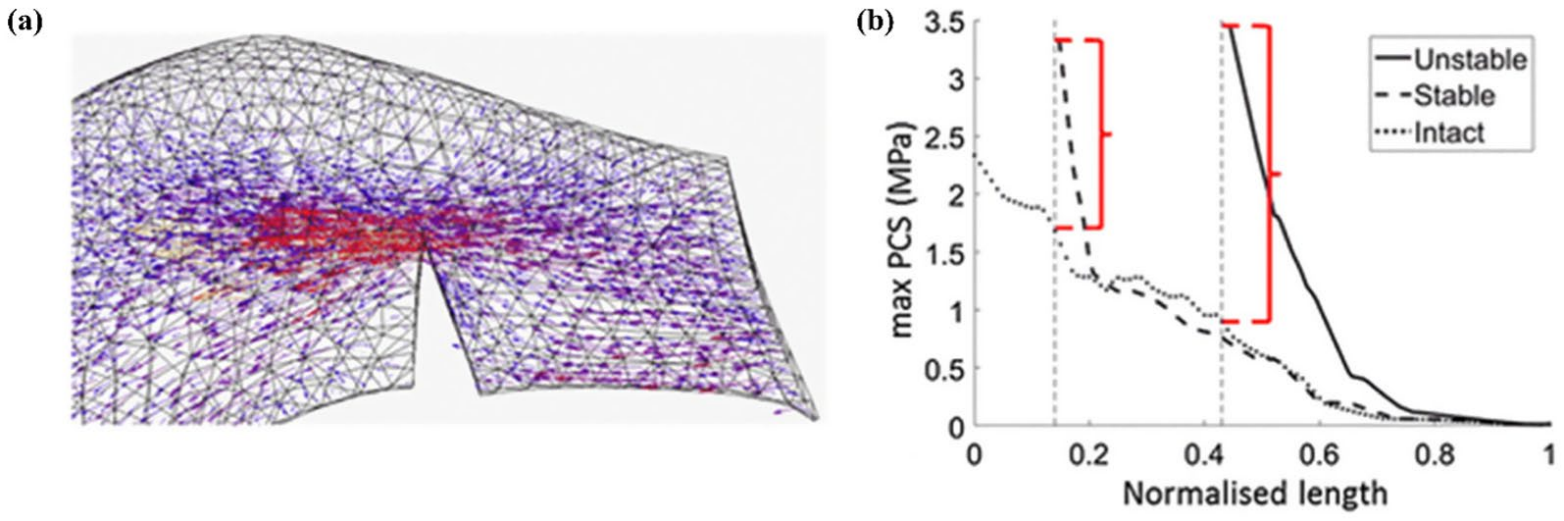
Kedgley et al. (18) finite element analysis tensor diagram. **(a)** Tensor diagram of unstable radial tear. **(b)** Maximum principal value of stress (max PCS) sampled from the inner (normalized length = 0) to the outer (normalized length = 1) rim for stable and unstable tears in the posterior segment of the medial meniscus for 0° knee flexion. Differences between the high stresses at the tear apex and the intact condition are indicated by the red brackets.

Kedgley et al. (19) first used finite element models to study the medial and lateral menisci biomechanics during knee flexion, providing an intuitive explanation for the results of clinical research at a deeper level of mechanical factors. As shown in Figure 9. The authors believe that radial tears of the meniscus destroy the continuity of the hoop collagen fibers, leading to a lack of hoop stress, reducing the bearing capacity of the meniscus, aggravating the wear of the articular cartilage, and causing the occurrence of osteoarthritis.

#### 4.1.2 Radial root tear

As a special type of meniscus lesion, the medial meniscus posterior root tears (MMPRT) account for 20% of all meniscal tears (104). MMPRT often occurs in the elderly population. MMPRT is closely related to meniscus herniation and osteoarthritis. Xu et al. (11) and Jiang et al. (40) conducted an in-depth analysis of different degrees of MMPRT. Jiang et al. (40) conducted a single-weight analysis of the length of the posterior root tear to the white zone, the red-white zone, the red zone, and the complete tear. The study showed that the posterior horn injuries of the medial meniscus could initiate combined injuries of the medial meniscus posterior horn (MMPH) and that of the medial meniscus body, and a combined injury of the MMPH and the lateral meniscus anterior horn. The hoop stress of the meniscus gradually decreases with the increase of the crack. It disappeared when the posterior horn is completely fractured, which explains why the joint space narrows when the meniscus posterior horn is injured in clinical practice.

**Figure 10:**
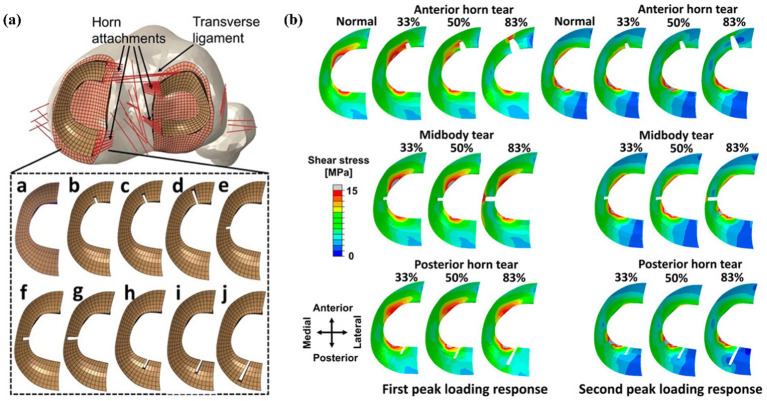
**(a)** Models of meniscus with different degrees of radial tears by Wang et al. (11). **(b)** Results of shear stress distribution on the medial meniscus involving a healthy knee and a knee with radial tears in the medial meniscus during the maximum weight acceptance and push-off.

Xu et al. (11) further analyzed the dynamics of partial and complete radial tears of the medial meniscus posterior root during the ISO gait cycle. Their results demonstrated that surgical repair significantly improved medial meniscal biomechanical function, supporting its current clinical preference. Radial tears may occur in any part of the meniscus. Wang et al. (12) used an FE-MS model to comprehensively analyze different degrees of tears (three tear widths: 33, 50, and 83%) occurring in the anterior horn, posterior horn, or midbody of the meniscus, as shown in Figure 10. Their findings revealed that total meniscectomy significantly increases the load on the joint compartment. Devaraj et al. (105) also corroborated this viewpoint in their review, concluding that partial medial meniscectomy is superior to subtotal/total medial meniscectomy.

### 4.2 Longitudinal tear

Longitudinal tears, characterized by their parallel orientation to the meniscal long axis, predominantly occur as traumatic injuries in younger populations. Pena et al. (17) made the first numerical calculations of meniscal tears and meniscectomy, significantly contributing to subsequent knee research. They compared different types of meniscal tears and corresponding meniscectomies. Dong et al. (23) proved this point in 2014. They concluded that longitudinal meniscectomy produced the greatest increase in meniscal peak stress and shear stress. Interestingly, they found that medial meniscus lesions and partial meniscectomy had little effect on the lateral compartment. The researchers proposed that after a meniscal tear or partial meniscectomy, the residual meniscus still has hoop stress to bear the load.

**Figure 11:**
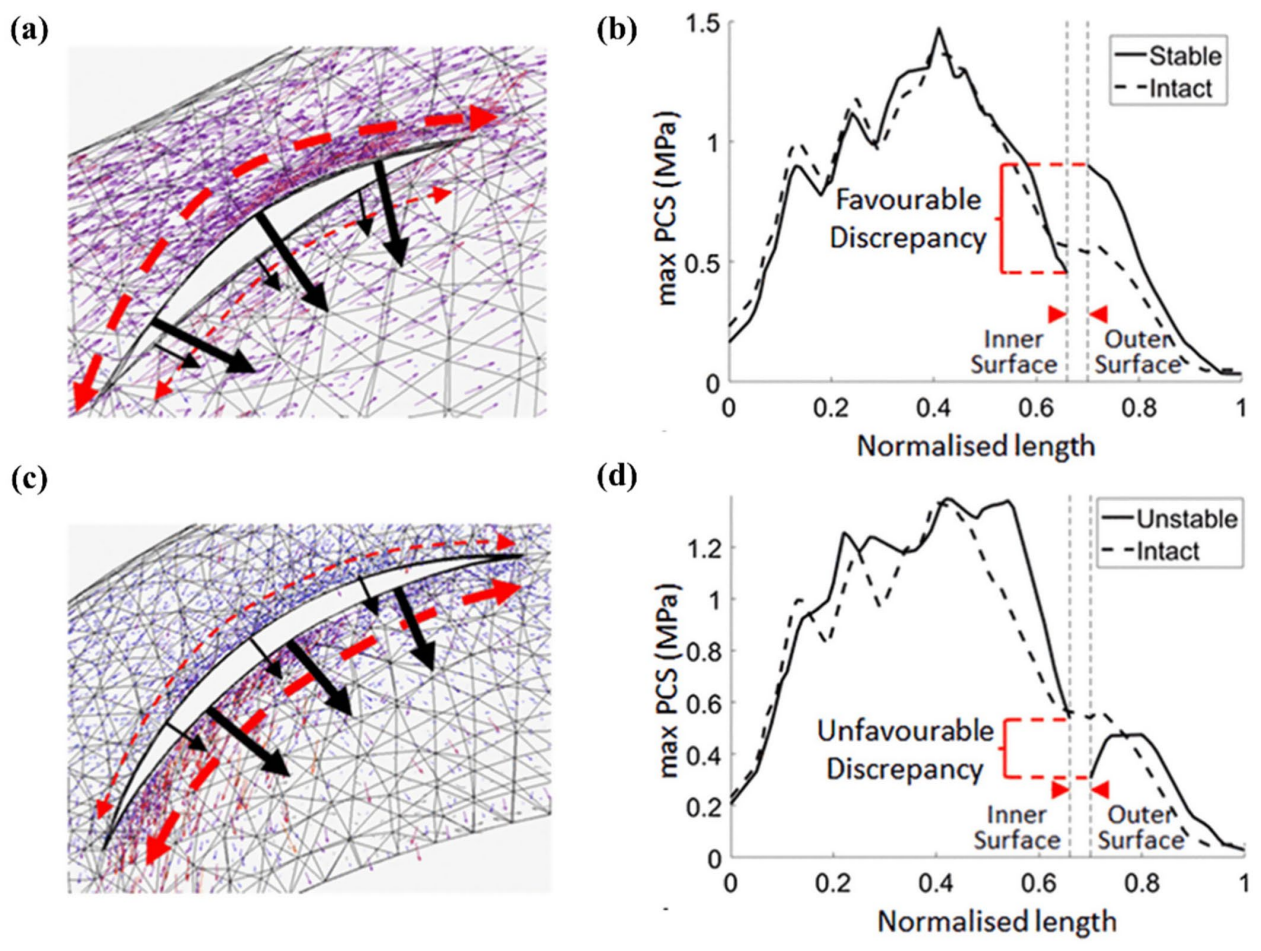
Kedgley et al. (18) finite element analysis tensor diagram. **(a)** Longitudinal stabilized tear of the medial meniscus at 30° of knee flexion. **(b)** Maximum principal value of stress (max PCS) sampled from the inner (normalized length = 0) to the outer (normalized length = 1) rim for longitudinal stable tears in the posterior segment of the medial meniscus at 30° of knee flexion. **(c)** Longitudinal unstable tear of the medial meniscus at 30° of knee flexion. **(d)** Maximum principal value of stress (max PCS) sampled from the inner (normalized length = 0) to the outer (normalized length = 1) rim for longitudinal unstable tears in the posterior segment of the medial meniscus at 30° of knee flexion. Dashed arrows represent hoop stress. Solid arrows represent the component of the stress tensor acting radially inwards. Thicker arrows represent higher magnitudes.

Stress concentration predominantly localizes at the tear edge of longitudinal meniscal injuries. Without intervention, these tears typically propagate bidirectionally under physiological loading. Zhang et al. (34) compared the longitudinal tears of the anterior and posterior horns of the medial and lateral menisci and found that the stresses were most apparent at the posterior horn of the medial meniscus, confirming the clinical research results. Currently, meniscus repair has become the first choice for longitudinal tears occurring in the red zone and red-white zone because of their rich blood vessels and the possibility of meniscus healing (106). Jiang et al. (40) studied the longitudinal tear of the posterior horn, which showed that the stress in the anterior horn of the medial meniscus increased, the body stress decreased, and the stress concentrated at the crack tip. Ardatov et al. (107) believed that longitudinal tears would lead to increased mise stress in the femoral and tibial cartilage, which was roughly the same as the stress distribution of radial tears. Kedgley et al. (19) intuitively explained the high failure rates of repairs of longitudinal tears of the medial meniscus in clinical practice from the perspective of the stress tensor, providing recommendations for conservative meniscus treatment. As shown in Figure 11. Notably, finite element modeling of longitudinal tear repair remains uninvestigated.

### 4.3 Root tear

**Figure 12:**
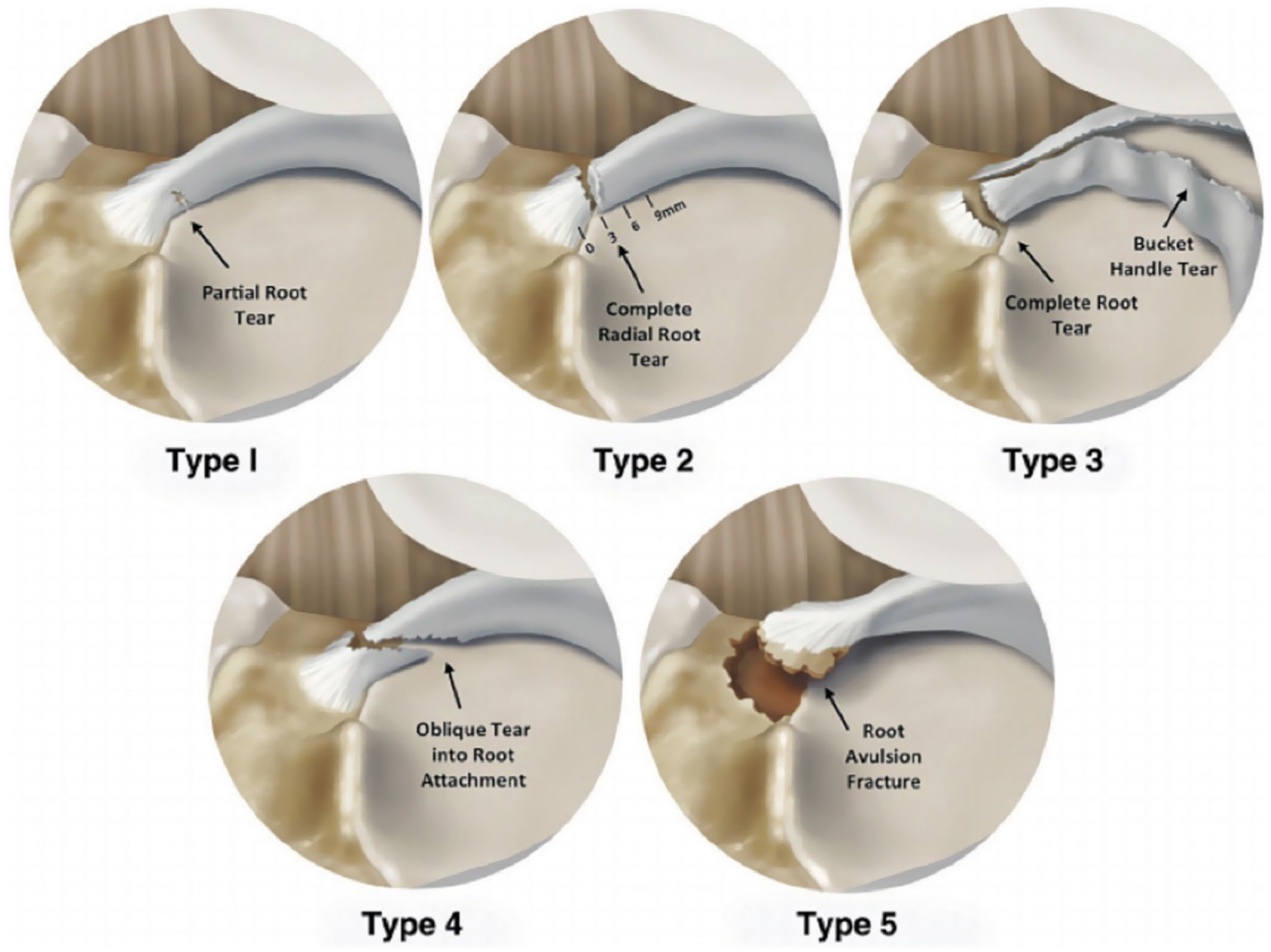
Diagram of classification of posterior tears of meniscus roots (109).

Meniscal posterior root tears result from trauma or degenerative joint disease. The medial meniscus exhibits significantly higher injury rates than the lateral meniscus, with medial root tears accounting for approximately 20% of all meniscal injuries (104). These tears disrupt hoop collagen fibers, causing hoop stress loss and accelerated osteoarthritis progression. In 2014, LaPrade et al. (108) proposed a classification method applicable to medial and lateral menisci posterior root tears based on the morphology of meniscal posterior root tears. As shown in Figure 12:

Type 1 (7%): Partial stable root tear;
Type 2 (68%): Complete radial tear within 9 mm of the bony root attachment;
Type 3 (6%): Complete radial tear within 9 mm of the bony root attachment;
Type 4 (10%): Complex oblique or longitudinal tear with complete root detachment;
Type 5: Bony avulsion fracture of the root attachment.

In the Radial tear section, the author has already described the radial tear of the posterior horn in detail. This type is also the most common type of posterior horn tearing. This section mainly describes the finite element study of other types of posterior root tears.

**Figure 13:**
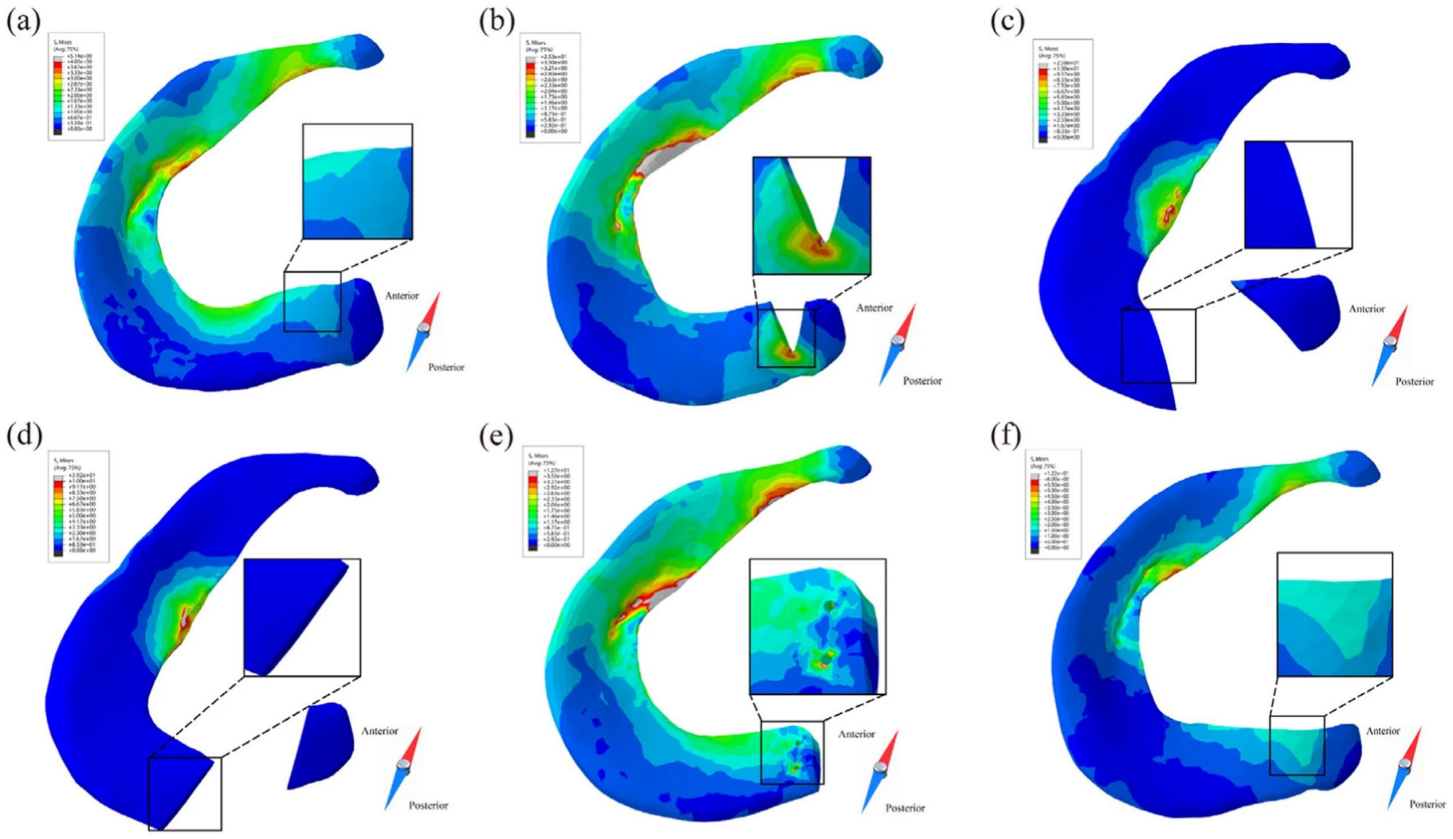
Stress distribution of posterior root of medial meniscus in different models at 10% of the gait cycle (10). **(a)** IK intact knee model. **(b)** PT partial tear model. **(c)** EOT entire oblique tear model. **(d)** ERT entire radial tear model. **(e)** MR meniscus repair model. **(f)** PM partial meniscectomy model.

Xu et al. (11) demonstrated that hoop forces still exist in the meniscus after partial meniscectomy. Stress after partial meniscectomy was higher than after meniscal repair, as showed in Figure 13. Chung et al. (109) showed the Lysholm and International Knee Documentation Committee (IKDC) Subjective Knee Form scores at the last follow-up in the meniscus repair group were significantly higher than those in the partial meniscectomy group. This suggests that meniscus repair has long-term value from a clinical and biomechanical perspective. Yang et al. (27) confirmed from gait analysis that meniscectomy significantly impacts joint stress under dynamic conditions.

Oblique or longitudinal tears of the posterior root of the meniscus usually result in the anterior horn bearing more load, leading to increased stress and a concentration of stress at the crack tip. When the tear is not complete, there is no obvious stress and displacement compared with the intact meniscus, which is similar to the result of a stable posterior root tear. Residual hoop stress may persist due to partial fiber continuity. Jiang et al. (40) believed that the hoop stress increased with the extension of the crack. Based on the research of Xu et al. (11), we thought that complete oblique tears and complete radial tears of the posterior root have similar mechanical behavior.

**Figure 14:**
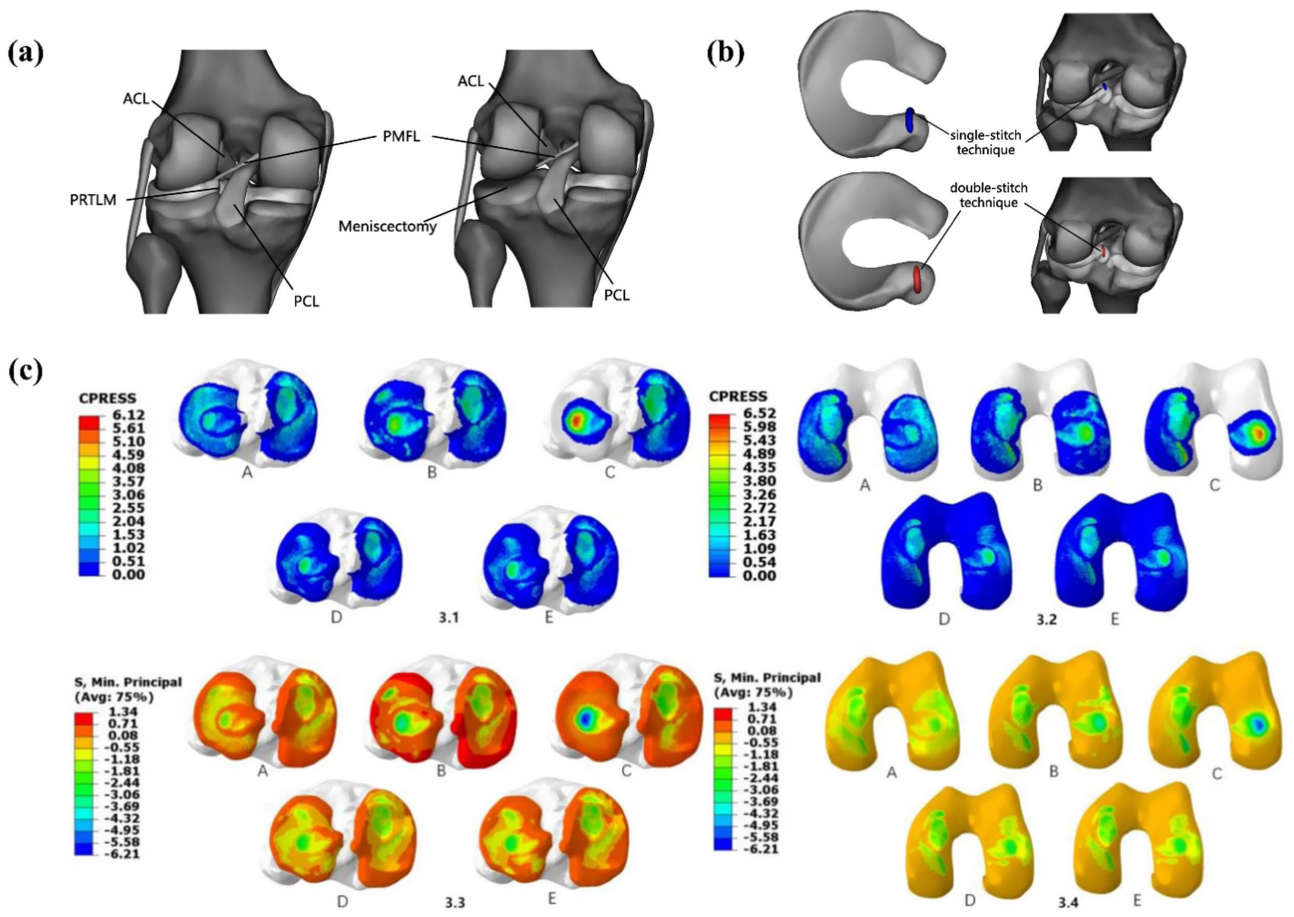
Surgical models established by Wang et al. (1). **(a)** posterior heel tear of the lateral meniscus and lateral total meniscectomy. **(b)** PRTLM junction reconstruction with single-stitch and double-stitch. **(c) (3.1–3.2)** Contact pressure distribution in the medial and lateral tibial and femoral articular cartilage under a 1,000 N axial compressive load. **(A)** Intact knee, **(B)** PRTLM, **(C)** lateral total meniscectomy, **(D)** attachment point reconstruction with the single-stitch technique, and **(E)** attachment point reconstruction with the double-stitch technique. **(3.3–3.4)** Contact stress distribution in the medial and lateral tibial and femoral articular cartilage under a 1,000 N axial compressive load. **(A)** Intact knee, **(B)** PRTLM, **(C)** lateral total meniscectomy, **(D)** attachment point reconstruction with the single-stitch technique, and **(E)** attachment point reconstruction with the double-stitch technique.

Wang et al. (2) studied three surgical techniques for lateral meniscus root avulsion, as shown in Figure 14. Compared with meniscectomy, single-stitch and double-stitch attachment reconstruction for the menisci posterior roots have good results. The double-stitch technique performs better than the single-stitch technique and significantly reduces joint stress. Steineman et al. (41) studied the positioning of the repair of posterior root tears of the medial meniscus and found that placing the repair at the anatomical point was the best choice. Biomechanical evidence confirms that even complete root tears retain partial load-bearing capacity via the meniscofemoral ligament (MFL), underscoring the clinical imperative for meniscal preservation (42).

### 4.4 Degenerative tears

Degenerative meniscal lesions demonstrate high prevalence. Large-scale cohort studies (110, 111) reveal that over 50% of degenerative tears remain asymptomatic, whereas 90% of osteoarthritis patients exhibit concomitant meniscal damage. These lesions primarily correlate with early-stage osteoarthritis, age-related changes, and systemic comorbidities, manifesting as horizontal, oblique, flap, or complex irregular tear patterns. Degenerative lesions often occur in the middle and posterior of the meniscus. Currently, meniscectomy has been widely used to treat degenerative meniscal tears.

Oblique tears are the most common type of tear, usually occurring in the white zone of the meniscus, and have almost no self-healing ability. Currently, the only option in clinical practice is partial meniscectomy. Dong et al. (23) studied radial and oblique meniscal tears at the same location. They found that oblique tears had longer tears in the area of maximum contact pressure, higher shear stress values, and more meniscus removed.

**Figure 15:**
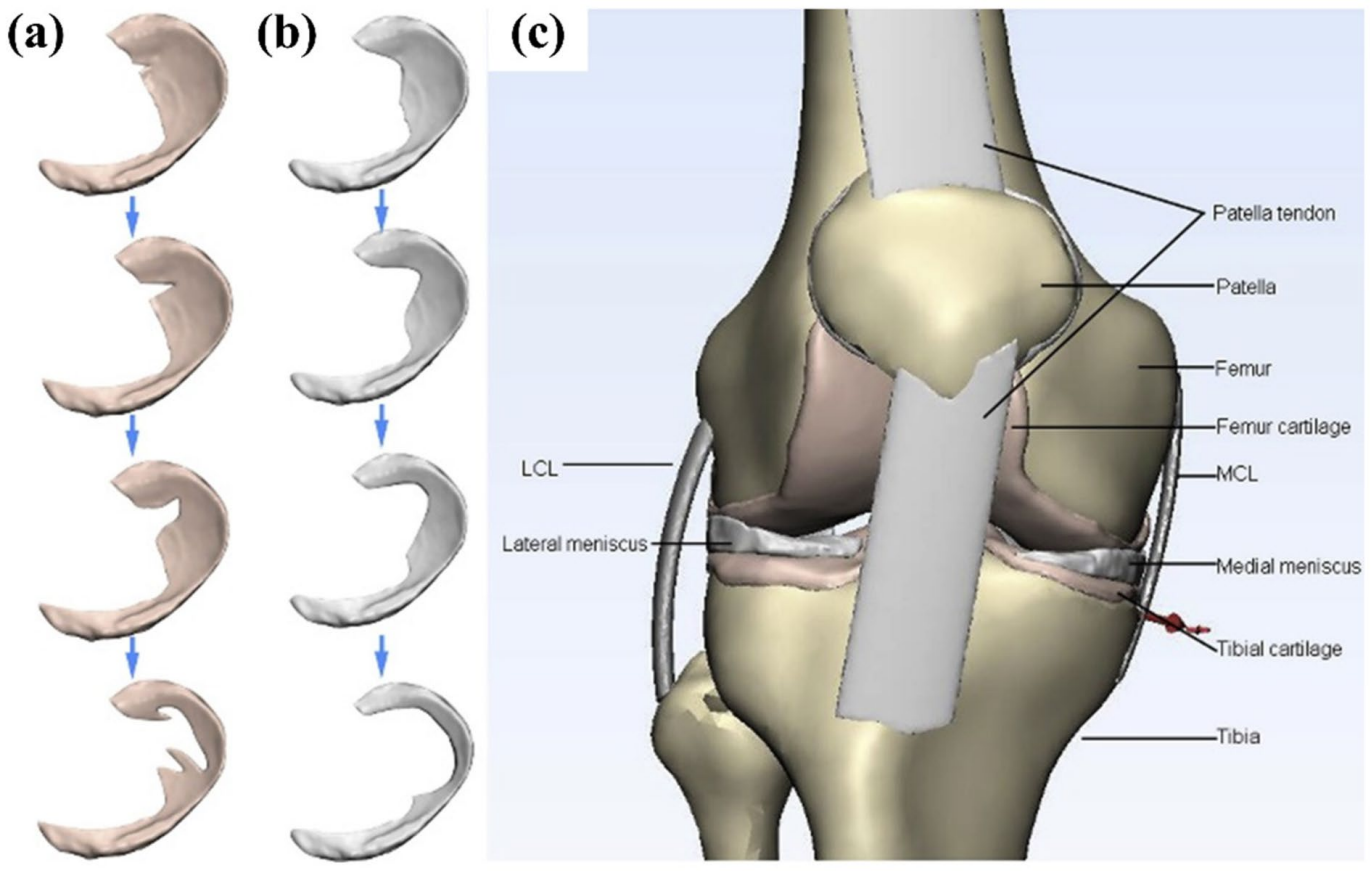
Diagram of the three-dimensional model used in the finite element simulation of Li et al. (36). **(a)** Aggravating degenerative medial meniscus tear models: from top to bottom: small oblique tear, big oblique tear, flap tear, and complex tear. **(b)** The related meniscectomy models of each meniscus tear. **(c)** A general view of the knee joint model. 3D, three-dimensional; FE, finite element.

Degenerative tears was a complex tear with irregular shape. As early as 2011, Bae et al. (20) established the lower limb model to study the degenerative tear of the meniscus. They established four models: intact, partial, subtotal, and total, and believed that partial meniscectomy has better results than subtotal and total resection. Zhang et al. (25) compared the mechanical changes of degenerative tears in the medial and lateral menisci and the corresponding meniscectomies. The medial meniscus showed more pronounced load redistribution, confirming its greater weight-bearing role. Degenerative tears with irregular shapes are more likely to have stress concentration at the crack tip during flexion, which explains why the occurrence of degenerative lesions lead to aggravation of the lesions. Li et al. (36) established four types of degenerative tears (small oblique tears, large oblique tears, flap tears, and complex tears), as shown in Figure 15. Meniscectomy led to further deterioration of meniscal extrusion as the degree of tear gradually worsened, and the results are consistent with the clinical presentation. They concluded that the rupture of the hoop fibers of the meniscus may be the cause of this phenomenon, which is consistent with the views of many researchers. In cases of more complex tears, a greater extent of meniscectomy may be required. However, this approach could lead to suboptimal clinical outcomes, suggesting that artificial meniscus implantation might serve as a viable alternative.

Horizontal tears caused by degenerative lesions extend from the inner free edge of the meniscus to the outer edge, dividing the meniscus into two layers with an extensive range of involvement. This easily leads to shear stress differences on the tear surface, which is unfavorable for healing and results in poor healing outcomes. Horizontal tears account for about 32% of meniscal tears (112). Jiang et al. (40) found that when the tear site occurs in the posterior horn of the medial meniscus, they cause increased stress in the anterior horn of the lateral meniscus. Chen et al. (113) believed that suture repair of horizontal meniscal tears is the best method, but when suture repair is difficult, resection of the upper lobe of the meniscus is also a reliable option. Li et al. (28) found that horizontal tears will cause large compressive stress in the medial femoral cartilage under static posture simulation, resulting in irregular biomechanical balance of the knee joint. Compared with other tears, horizontal tears have a wider range and will cause more significant compression and shear stress, but Xu et al. (23) denied this view. They used the “hoop strain” theory proposed by Krause et al. (114) to partially explain this. For horizontal or longitudinal meniscal tears, the torn meniscal fragments still maintain the majority of the hoop strain capacity to perform their load-bearing functions.

### 4.5 Bucket-handle tears

A meniscal bucket-handle tear, typically originating from the posterior horn and extending to the body or anterior horn, involves extensive tissue damage. These tears frequently coexist with anterior cruciate ligament injuries and may induce joint locking.

Surgical repair remains the preferred treatment. For irreparable tears, subtotal meniscectomy is typically performed due to the lack of consensus on alternative approaches. Devaraj et al. (115) reported that bucket-handle tears result in a significant increase in the maximum principal stress at the crack tip, along with a 43.18 ± 27.59% rise in meniscal contact stress. These elevated stress levels may ultimately lead to complete meniscal rupture. Notably, finite element analysis of repaired meniscal bucket-handle tears has not been conducted, likely due to the technical challenges in developing such computational models.

### 4.6 Discoid meniscus

Discoid meniscus, a congenital meniscal deformity characterized by inferior structural integrity, exhibits a higher propensity for tearing and subsequent osteoarthritis (37, 116). Preserving both the width and anatomical shape of the discoid meniscus to optimize biomechanical function remains a clinical challenge. Mochizuki et al. (117) demonstrated that a discoid meniscus with a remaining width less than 7 mm exacerbates cartilage damage. Some scholars (118) also suggested that the meniscus width should be retained within the 6–8 mm range, which is also currently used in clinical practice. However, Yokoe et al. (37) advocated for maximal meniscal preservation during surgery. Computational studies by Liu et al. (47) on 10 lateral meniscal models identified 8–10 mm as the biomechanically optimal width. The latest research evidence further indicates retaining more than 55% of the meniscus volume is necessary to prevent a significant increase in joint stress (119).

Individual-specific reasons lead to differences in research results among different scholars. Ultimately, the evaluation should be based on the patient’s condition. Given the irreversible nature of meniscectomy, collaborative efforts between clinicians and researchers are essential to develop optimized surgical strategies.

## 5 Discussion and future perspectives

### 5.1 Discussion

This study provides a narrative review of the construction of finite element models for meniscal tears in the knee and finite element analysis of meniscal tears and surgical techniques. This study aims to provide researchers with more reasonable finite element models, evaluate the biomechanical properties of meniscal tears and related surgical techniques, and provide more systematic clinical research to further improve surgical techniques.

Finite element analysis (FEA) of the meniscus primarily investigates common tear patterns and surgical interventions, with stress and displacement serving as key evaluation metrics. These studies explain the clinical manifestations of meniscal tears through biomechanical mechanisms. The results demonstrate that the rupture of hoop fibers generates increased hoop stress, which is a major contributor to tear propagation. Interestingly, the “stress difference” induced by tears does not always have detrimental effects, offering theoretical support for certain surgical techniques. This review assesses the outcomes of different surgical techniques. Previous studies have shown that meniscectomy leads to elevated contact stress, whereas preserving meniscal tissue and opting for repair strategies yield better clinical outcomes.

In order to obtain accurate numerical results, the researchers performed the following specific work throughout the process, it must be followed:

1. Establish an actual geometric model of the basic knee joint, including bones, ligaments, meniscus, cartilage, muscles, and tendons.
2. According to the research needs, establish knee joint models with different component injuries and activity modes， and determine the real lower limb alignment.
3. A mesh convergence study ensures that the numerical results remain independent of element size.
4. Set reasonable boundary conditions and loads to simulate correct physiological activities.
5. Select the correct material properties to describe the mechanical properties of each knee joint tissue accurately.

There are still many limitations in the finite element analysis of the knee joint. The main ones include the following points:

1. In most literature studies, FE-MS was not implemented, which significantly affected the accuracy of gait analysis. However, accurately incorporating muscle force is a very challenging task.
2. The soft tissue structure does not consider biphasic behavior and cannot truly describe the accurate mechanical properties of the knee joint tissue. The soft tissue of the knee joint needs to be combined with the material multiphase model, which is the future development trend of the finite element model from macroscopic to microscopic analysis modeling.
3. The incorporation of ligaments and other soft tissues introduces significant nonlinearities, rendering dynamic analysis of knee joint models that include solid ligament representations particularly challenging.
4. Few researchers have considered the effects of the joint capsule and synovial fluid around the knee joint. The model does not perform fluid–structure interaction (FSI) analysis. It is impossible to clearly understand the role of synovial fluid from a mechanical perspective.
5. here is almost no literature analyzing the impact of meniscus repair on knee biomechanics of the knee joint, and modelling difficulty is the main reason, in our opinion.
6. The current study only involves patient-specific modeling，the current model’s limited coverage of individual anatomical variations (e.g., discoid meniscus), as well as the impact of missing biomechanical parameters (e.g., synovial fluid viscosity) on the results.

Based on the literature and data collected, no perfect model exists, including the entire lower limb structure, three-dimensional models of all tissues, synovial fluid analysis (fluid–structure interaction), and biphasic modeling of soft tissue.

### 5.2 Future perspectives

Recent advancements in knee biomechanics suggest that multiscale modeling represents a pivotal direction for future breakthroughs. Traditional finite element (FE) models of the knee meniscus have evolved from macro-mechanical analyses to micro-mechanical investigations, enabling more precise simulations of meniscal behavior under complex loading conditions (e.g., compression and rotation). The incorporation of multiscale constitutive model, such as the Holzapfel–Gasser–Ogden (HGO) formulation, has significantly improved the accuracy of macroscale mechanical predictions. A critical advantage of multiscale modeling lies in its ability to bridge tissue-level damage mechanisms with permeability dynamics, particularly when integrated with biochemical signaling and mechanobiological coupling. Such integration offers novel insights into meniscal repair strategies. Combining finite element analysis with the design of 3D-printed meniscus scaffolds—parameterized by porosity and fiber orientation to match natural tissue mechanics—provides a promising approach for artificial meniscus development (120, 121). The use of multi-physics field models with fluid–structure interaction (FSI) is crucial to understanding the mechanism of meniscus degeneration and provides a meaningful mathematical model for clinical use.

## 6 Conclusion

This review summarizes the numerical analysis of clinical meniscal tears and corresponding surgical techniques (repair, meniscectomy, etc.) through finite element methods, aiming to provide researchers with more reasonable FEA models and assist surgeons in selecting techniques with lower stress distribution and reduced risk of post-operative degeneration. Currently, meniscal repair remains the preferred treatment with proven clinical efficacy. FEA can predict the outcomes of different surgical techniques for various tear types, supporting personalized treatment planning and further refinement of surgical techniques. Finally, we propose high-accuracy finite element models as reliable biomechanical evaluation tools for optimizing meniscal repair strategies.
